# "The Hospital" Nursing Mirror

**Published:** 1897-01-16

**Authors:** 


### "The Hospital" Nursing Mirror.

Being the Nursing Section of "The Hospital."

[Contributions for this Section of "The Hospital" should be addressed to the Editor, The Hospital, 28 & 29, Southampton Street, Strand, London, W.C., and should have the word "Nursing" plainly written in left-hand top corner of the envelope.]

### News from the Nursing World.

### THE QUEEN'S COMMEMORATION.

The Countess of Strafford, the Countess of Selborne, Lady Lucy Hicks-Beach, Lady Rothschild, Mrs. Henry Grenfell, Mrs. Beerbohm Tree, and Miss R. Paget have joined the Executive Committee of the fund for extending the work of the Queen's Nurses.—At a meeting held at Salford, under the presidency of the Mayor, it was resolved to commemorate the sixtieth year of Her Majesty's reign by some permanent memorial; and a suggestion to establish a branch of the Queen's Jubilee Institute for Nurses was very favourably received. A committee has been appointed to consider the matter.

### THE PROTECTION OF YOUNG GIRLS.

The Princess Louise Home and National Society for the Protection of Young Girls does an excellent work in caring for and training girls who, whether orphans or otherwise, are friendless and unprovided for. Some 1,600 girls have been, through its agency, rescued from evil surroundings and trained for domestic service, and at the end of last year there were 107 girls in the home at Kingston Hill, the largest number ever in training at one time. The reports on the school work by the Government inspector have been invariably satisfactory, and the maximum grants have been allowed in every subject of examination. The laundry, built in 1892, has proved an entire success, the earnings therefrom being a considerable help towards the funds of the society. Now, a new schoolroom and additional washhouses are urgently needed, and for this the committee are appealing to the public for aid. The Princess Louise, Marchioness of Lorne, after whom the home is named, takes keen personal interest in its welfare. Subscriptions and donations should be sent to the secretary, at the office, 32, Sackville Street, Piccadilly.

### ADDENBROOKE'S NURSES' HOME.

The recently-built nurses' home at Addenbrooke's Hospital is at present unconnected in any way with the hospital proper, but a winter's experience of the risks and inconveniences of crossing from home to hospital, and *vice versa* in wet and cold weather has made apparent the necessity for a covered passage between the two buildings. This is now to be put up; a friend of Miss Cureton's has promised to provide the funds, and a plan has been drawn up by Mr. Macalister, so that the work can be proceeded with without delay. It is a most necessary addition to the comfort of the home. Nurses are none too careful of their own health as a rule, and it is just these short exposures in bad weather when it seems "not worth while" to put on a shawl or thick shoes that result in colds and chills, from which serious consequences may follow.

### NURSING AT THE BOW INFIRMARY.

By thirty-five votes to twenty-two, the Guardians of the City of London Union have rescinded their recent resolution to abandon the scheme of reorganisation of the nursing department, so far as it related to the training of probationers, urged upon them by the Local Government Board. A great deal of time has been wasted and nonsense talked over this matter by those who demonstrated nothing more clearly than their own incapacity to understand the question of nursing at all. When the new scheme has been put into operation, and the whole nursing department brought into line with other modern infirmaries, the Guardians will no longer be beset by their present difficulty of filling vacancies on the staff.

### METROPOLITAN HOSPITAL, KINGSLAND ROAD.

An excellent entertainment was provided for the patients at this hospital on Thursday in last week. It was held in one of the empty wards, and heartily enjoyed, the programme including contributions from numbers of well-known and popular artistes. There were songs of all kinds, humorous and otherwise, trios by the Sisters Hawthorne, recitations by Mr. Lewis Waller and Mr. Frank Celli, "Ventriloquism Up-to-Date" by Mr. Fred Russell, and imitations of popular actors by Mr. Arthur Faber. The hospital still looked very festive in its Christmas dress, and with its staircase and wards decorated with fairy lights and pretty plants, and the evening was altogether a great success.

### CONGLETON COTTAGE HOSPITAL.

Miss Wagnell, matron of the Congleton Cottage Hospital, was presented this Christmas by a number of lady subscribers with a purse, amounting to £12 10s., in evidence of their "high esteem," and many pleasant and gratifying things were said of her by Lady Shakerley, who made the presentation, and other ladies. Miss Wagnell has held the post of matron for twelve years. Christmas was celebrated at the hospital by a tea and magic-lantern entertainment, much enjoyed by the patients, and a number of useful presents were received from friends in the neighbourhood in the practical shape of game, milk, puddings, and plenty of cake and fruit for the tea.

### IMPROVED NURSES' QUARTERS.

The Sheffield Board of Guardians, at a recent meeting, adopted a recommendation of the Hospital Committee regarding the urgent necessity for providing additional and more convenient accommodation for the nurses. A sub-committee has therefore been appointed to consider and report on the "best way of increasing the accommodation, either by additions to the present Nurses' Home, or by the provision of a new home elsewhere."

### A HOME OF COMFORT AT SOUTHSEA.

A home for the dying has just been started at Dowran, Victoria-grove, Southsea, under the management of the Mother Superior of the Diocesan Deaconess Home, intended for the reception of better class patients, clergy, governesses, nurses, and clerks, unable to pay fully for the nursing and care needed in their last days. It is specially desired that the home shall be for the comfort of those who "need loving care after, it may be, years of work for others." There is accommodation for twelve patients "in the last stage of illness, who are without relations and friends able to nurse and give them the care they need." They are expected, when possible, to pay towards their support in the home according to their means. Applications for admission to be made to the Mother Superior, St. Andrew's Home, Portsmouth.

### WORKHOUSE NURSING AT HEREFORD.

IT has evidently not penetrated the intelligence of the Hereford Board of Guardians that trained nurses are not engaged to do the work of charwomen or ward maids, but to see to the well-being and proper tending of the sick in the infirmary wards. At a recent meeting of the Board "it was reported that the two nurses had resigned, and it was stated that in future they did not intend to have certificated nurses, because they objected to domestic work." One more instance of guarding the rates at the expense of the unfortunate paupers.

### KETTERING AND DISTRICT GENERAL HOSPITAL.

THE committee of the Kettering General Hospital have every reason to rejoice over the really wonderful success of the bazaar held this autumn in aid of the funds. At a special meeting last month a cheque for something over £3,000 was handed in as the net result of this effort. A pleasant recognition of the energetic services of Mr. Frank Berrill, the secretary of the hospital, took the form of a presentation to him of a silver inkstand from the stall-holders and helpers at the bazaar, as a small token of their appreciation of his services on that occasion.

### NEWS FROM IRELAND.

MISS SARAH HAMPSON, the retiring lady superintendent of the Rotunda Hospital, Dublin, is intending to start a private hospital and nurses' home in Dublin.—Miss Susan Beresford, who has recently resigned her post as lady superintendent of the City of Dublin Hospital, is to be presented with a testimonial by a number of her friends in Dublin. She has already received from her "past and present staff nurses" a beautiful parting gift in the shape of a "grandfather's" armchair.

### SOCIETY OF TRAINED MASSEUSES.

THIS society was started in 1894, and has rapidly attained an important position amongst medical men and trained masseuses. This year a statement of its objects and rules has been issued, accompanied by a long list of distinguished medical patrons. Briefly, the society was established (1) to improve the training and qualifications of masseuses, and organise an independent examination for them; (2) to establish a registry for members, and form a centre of information for the public on matters connected with massage; (3) to arrange lectures, provide a good medical reference library, and afford opportunities for the discussion of subjects of interest and importance to masseuses; and (4) to provide an organisation to which members would have a right to apply for advice and help in professional difficulties. Such a society fulfils a most useful mission, both to the medical profession, who thus know where to apply for trustworthy and efficient workers, and to the large number of women who have adopted massage as a profession, and who, through the society, can obtain instruction, advice in difficulties, and employment. The society is worked in connection with the Trained Nurses' Club, 12, Buckingham Street, Strand, where all information can be had from the secretary.

### BARRY NURSING ASSOCIATION.

A VERY serious and lamentable report of the financial condition of the Barry District Nursing Association was received and discussed at a recent meeting of the executive, Major-General Lee, hon. treasurer, stating that the association was practically in debt to the amount of some £300 at the end of the year. In view of the fact that keeping up the accident ward would entail at this rate a heavy loss each year, a meeting of subscribers was decided upon for the 21st inst., when a resolution will be put to the effect that the Nursing Association, finding the maintenance of the accident ward too severe a tax upon their resources, the Barry Urban District Council be asked to take over the control and care of the ward.

### DEATH OF AN ENGLISH NURSE FROM PLAGUE.

So far Europeans have seldom fallen victims to the plague, and during the Hong Kong outbreak their immunity was remarkable. At Bombay, however, several English people have been attacked, and the death of Dr. Manser has been quickly followed by that of Nurse Joyce, the English nurse who attended him. This sad news has caused much uneasiness in England amongst those who have relatives and friends in Bombay.

### ACCIDENT TO AN ARMY NURSING SISTER.

MISS WILDMAN'S many friends in England will be truly sorry to hear that on her way back to India, after a year's furlough, Miss Wildman met with a severe accident, shortly after leaving Queenstown, on Christmas Eve. The weather was stormy, and the lurching and rolling terrific. Miss Wildman, in attempting to reach her cabin, was thrown down at the top of the companion ladder, and sustained a compound fracture of the left leg. Notwithstanding the severity of the gale and the movement of the ship, Surgeon-Captain Jackson, A.M.S., set it most successfully, and she was removed to the floor of the music saloon, where she remained till she was put on shore at Gibraltar on December 28th, and removed to the Station Hospital, where she will remain for some time. Owing to the unsteadiness of the ship, she suffered much at first, but is now progressing favourably.

### SHORT ITEMS.

PRINCESS FREDERICA'S Convalescent Home at East Molesey has been closed, and we understand that the house and furniture are to be sold.—Lady Percy is taking much interest in the formation of a County Nursing Association in Northumberland. She has accepted the office of president, and is interesting herself personally in the scheme.—The *Westminster Gazette*, in speaking of the Guy's Hospital Nurses' Cycling Club, remarks: "The pretty uniforms and streaming ribbons of these ladies will add an agreeable charm to the rather sombre appearance of metropolitan thoroughfares"! Comment would be superfluous!—Mrs. Keogh, for thirty years a nurse at Guy's Hospital, and for twenty-five of these surgical night nurse, has just retired upon a well-earned pension.—Among the probationers at the New York Hospital Training School for Nurses is a Japanese lady, Shidzu Naruse Sen, a graduate nurse of the Missionary Hospital at Doshishi.—The Queen's Commemoration Fund at Belfast has reached the sum of £47,000, and a meeting has been called by the Lady Mayoress to aid in raising it to the desired £100,000.

### Hygiene: For Nurses.

By John Glaister, M.D., F.F.P.S.G., D.P.H.Camb., Professor of Forensic Medicine and Public Health, St. Mungo's College, Glasgow, &c.

### XXXIX.—DISINFECTION OF ROOM (*continued*)—GERMAN AND FRENCH MODES—SANITARY LAW.

After the room has been subjected to the influence of sulphurous acid or chlorine gas for 18 or 24 hours, it may be entered by a person wearing a damp flannel cloth over mouth and nostrils, who should then throw wide open the window, and the damper of the grate. The room should then be left until the irritating fumes have sufficiently disappeared to enable persons to work therein. Whereupon the floor, woodwork, and furniture ought to be freely washed with 1 in 4,000 corrosive sublimate solution, the wall-paper stripped, and the ceiling lime-washed, the windows remaining wide open all the while, night and day.

The other main line of treatment is directed against the deposited dust of the room, on the principle that the infective microbes and spores are there located. In Paris, disinfection of the room is carried out by spraying the apartment, including the furniture, with a fine spray of 1 in 1,000 corrosive sublimate solution, which is ejected from a machine called a *pulverisateur*, not unlike in appearance to a small fire-extinguisher. It is either carried on the back of the operator, or is set on the floor on wheels. The workman, during the operation, wears an overall uniform, which, when the work is done, is taken off, packed in a small tin case, and returned to the disinfecting station. The charwoman does the rest.

In Berlin a dry method of dealing with the deposited dust is first carried out. The material used consists of chunks of ordinary German bread, forty-eight hours old; and the workman, using the crust side of the bread as a handle, rubs systematically the walls and ceiling of the room. The dust becomes detached from the rubbed surface on to the bread, which is then burned. The walls are next sprayed with a 2½—5 per cent. solution of carbolic acid, the floor, woodwork, and furniture with the same solution, and thus room disinfection is completed.

These appear to us to be more rational and more effective modes of disinfection than is ours of aërial disinfection. The cost may be somewhat greater, but that is not worthy consideration when efficiency is our object. The chief objection to the free use of corrosive sublimate solution is its possible harmful effect on those who work with it, and on the inmates of the room after rehabitation. Experience in Paris has, however, shown that if the workmen are instructed as to its nature and effects, such risks are reduced to a minimum; and no harmful effects on the inmates have been recorded after reoccupation of a room so treated.

### Sanitary Law.

Ignorance of the law is no plea for contravention of it, therefore every householder and every nurse should be acquainted with at least its general principles. A district nurse may have many opportunities of combatting the ignorance which prevails in this respect, and will thus become of great assistance to the sanitary administration. Most of the sanitary legislation that presently is on the Statute Book has been passed by Parliament during the last fifty years, and it is to be deplored that such enactments do not all equally prevail in the constituent parts of Britain and Ireland. At the same time, the same general principles of sanitary law obtain all over the Kingdom. The main provisions of all these Acts are intended to conserve the public health by the removal of anything which may imperil it, and to prevent the origin and the spread of infective diseases. Our attention will be especially directed to the latter. In none of the Public Health Acts is there any definition of what constitutes an infectious disease, and up till 1889, when the Notification of Infectious Diseases Act was passed, there was no attempt even to give a list of these diseases. In that Act—a permissive Act—the diseases scheduled were small-pox, cholera, diphtheria, membranous croup, erysipelas, scarlet fever or scarlatina, and typhus, typhoid, enteric, relapsing, continued, and puerperal fevers. It will be observed that some, as measles, whooping-cough, chicken-pox, influenza, and mumps, are omitted from the list, but it must be remembered that the provisions of the Public Health Acts equally apply to these as to the former list. The provisions of Public Health Acts deal (1) with the infective person; (2) with the infected place; and (3) with infective things. They may, therefore, be considered in this order.

I. In Respect of the Infective Person.—It is forbidden, under a penalty, for any person suffering from an infectious disease (1) to wilfully expose himself in any public place or conveyance, without taking proper precautions against spreading the disease; (2) to enter a public conveyance without giving the owner, or person in charge, due notice, when it shall be within the option of the owner, &c., to refuse, but who, if he conveys such person, is entitled to be indemnified by said person against loss incurred thereby, and for expenses of disinfection; (3) to expose any person who suffers from an infectious disease in any public place, &c. All these provisions are most necessary and every sanitary authority ought to make examples of all transgressors. On one occasion we found a person who was convalescent from small-pox, but whose body was still marked with "crusts," promoting his convalescence by journeying to and from the city and one of the suburbs of Glasgow in an omnibus. It is needless to say that he received smart punishment.

Removal of Infective Persons.—Where an isolation hospital has been provided, any person suffering from such a disease who is without proper lodging or accommodation, or who is lodged in a room occupied by others besides those in attendance, or who is on board any ship or vessel within three miles of the coast may, on a warrant of a magistrate, be removed to such hospital. In like manner a person in a common lodging-house may be so removed. Again, persons who have occupied or who occupy the same room as an infective person, may be removed to other suitable accommodation provided for them by the Sanitary Authority. These provisions are to be interpreted by the existence or non-existence of means of efficient isolation and facilities for disinfection. It is forbidden for anyone, who himself is suffering from an infectious disease, or who himself directly, or by others indirectly, has come in contact with an infective person, to be concerned in the production, distribution or storage of milk; and in London an infected person is forbidden to follow or take part in any occupation which is connected with any food-production, the products of which are likely to spread the disease. All of the foregoing provisions apply to the living infective person only. What of the dead? No precautionary or prohibitory provisions exist in any of the Public Health Acts regarding the removal, in a public conveyance, for burial of an infective dead body. In England and Ireland, however, the Infectious Diseases (Prevention) Act requires that due notice shall be given to the owner or driver, and the person giving notice shall be bound to pay the costs of disinfection of the vehicle used. Where a public mortuary has been provided, and where any dead body in any room is certified to be inimical to the health of the occupants of the room, such body, on a warrant, may be removed to the mortuary. Again, in view of the Irish custom of "waking" the dead, and of the dangers which may arise therefrom, such a practice is prohibited in certain local Acts (as Glasgow), when the death has resulted from an infectious disease.

II. In Respect of the Infective Place.—As we have already seen, public conveyances used for the carrying of the infective sick must undergo disinfection. In like manner, the room occupied during an infective illness must also be disinfected—(1) either at the expense of the householder, or (2) from the public rates. While it is common for the latter mode to be carried out, it ought to be remembered that the duty, according to the Acts, devolves upon the householder. At the same time, no sanitary authority which does not provide the necessary facilities and materials can ever expect disinfection by private persons to be thorough. Again, any person who knowingly lets any house, room, or part of a house or shop which has not been properly disinfected after occupation by an infective person, or who withholds such information on being asked, or who makes any false statement regarding the same, shall be liable to heavy penalties.

III. In Respect of Infective Things.—Anyone who, without previous disinfection, knowingly gives, lends, sells, transmits, or exposes any bedding, clothing, rags, or other things which have been exposed to infection from infectious disorders, shall be liable to a penalty. This does not apply to articles transmitted with proper precautions for purposes of disinfection. The following may be taken as illustrative cases, viz., (1) trafficking in anything from an infected room; (2) carrying infected articles through the public streets, or sending such in a public conveyance; (3) washing infected clothing in a washhouse which is common to other houses, or sending such clothing to a public laundry; (4) making fabrics for warehouses or for private persons in an infected house, and carrying the same to the warehouse or private person when made; (5) pawning infected articles; (6) selling food of any kind in a shop attached to a dwelling-house in which infective disease is present; and (8) transmitting infected bedding or furniture to upholsterers or public cleaning establishments.

The term "other things" would include infected rubbish, such as excreta, poultices, wall-paper, or vomited matters cast into a receptacle in a public place without previous disinfection. But to make this perfectly clear the Infectious Diseases (Prevention) Act contains a special provision to the above effect.

In respect of school attendance, the Glasgow Sanitary Acts make it an offence for a parent or person in charge of any child who is suffering from infective disease, or who resides in a house where such disease exists, or has existed within six months, to knowingly or negligently permit such child to attend school without producing a medical certificate which states that the child is free from disease and infection, and that the house has been properly disinfected; and it is made equally penal for a teacher or person in charge of any school to knowingly permit such a child to attend school.

### A Nurse's Visit to Texas

II.

The ranch was over 1,300 ft. above the sea level, and our little wooden house stood on a dry, bleak-looking hill, a very healthy position, free from frosts and ague, but a trying one in the high winds of winter. Often at night I was aroused by the noise of the storm; the wind, getting under the broad verandahs, seemed every now and then to give the house a little lift, as if it meant to carry it away bodily, and I lay in bed wondering how long such a frail shelter could withstand its force.

We saved all the soft water we could collect in a barrel, ready for washing day, and if a heavy rain came on in the night we used to get up and fetch out tubs and pails to catch the water as it streamed off the roof; besides that, the board walls having cracked with the heat and dryness of the climate, the rain soon found its way into the rooms, so pictures and anything spoilable had to be moved into a safe place.

We had a well of very good drinking water close to the house. You would not think there could be a drop of moisture in such a bare, dry hill, but before the house was built it was necessary to make sure of a water supply, so a man came with a divining rod, and right on the hill top the rod indicated a strong spring; they bored and came upon it at the depth of 100 ft., and the water generally stood 60 ft. deep in the well, which was only 5½ inches in diameter.

We were encircled by hills at a little distance on all sides except the north-east, and there, through a gap we could see so far over the lowlands that in the distance they looked so like the sea it was possible to fancy the waves moving.

I soon got into the routine of ranch life; each day it was much the same. Henry, being an indulgent husband and brother, got up first, lit the fire, put on the kettle, and went off to the pens to see the animals, while we got breakfast ready. We lived principally on bread, white and corn (the latter made from ground maize), bacon, boiled rice, oatmeal porridge, vegetables from our garden, dried apple chips, molasses, milk, tea, and coffee. Fresh meat was a luxury. When we had that it was generally in large quantities. If, for instance, a neighbour meant to kill a "beef," as they called the bullock, three or four families would promise to buy so much. Then we would roast some, salt some more, and the rest was chopped, seasoned, stuffed into calico bags, and dried in wood smoke, to serve as sausages for future use. When there was enough milk I used to make a little butter.

Breakfast over, the men went to outdoor work, while we did the housework, and then sat down to our sewing. There was always plenty of that to be done. The sun rots the clothes, and makes the men's shirts wear out very fast. Dinner was at noon, and after that was put away we rested a little while before going back to our sewing. Then, when it grew cool enough, we went out for a stroll or to look after the garden. Supper at sunset was the next event; then we read, worked, and talked till bedtime.

Of course, you understand there were no domestic servants. Even if the settlers could afford to keep them, which they cannot as a rule, it would be difficult to get them, and, if they succeeded, the "hired help" would probably be more plague than profit. The nearest approach I met to a house servant in that part was a negro, one-legged and half-witted, whom an Englishman found in one of the towns, being made to dance in the streets for bread. So he brought him home and set him to nurse his baby, and now that the children are all grown up, "Jim" still stays on, doing little odd jobs about the house.

We did all our own work, washing and ironing, cooking, baking, dressmaking, and even shoe-mending, for when you have to drive seventeen miles over a bad road to the nearest town, you naturally do not want to go oftener than you are obliged.

### Presentation.

On Christmas Day, Miss Rickards, the matron of the Sheffield General Infirmary, was presented by the nursing staff with a handsome silver-mounted writing case, and with glove and handkerchief case *en suite* from the servants. This was a farewell gift, for Miss Rickards is shortly leaving the infirmary, where she has held the position of matron for eight years.

### The R.B.N.A. "Irreconcilables" and Mental Nurses.

(A SKETCH.)

A MEETING was held in the Lower Hall, St. Martin's Town Hall, W.C., on Thursday, 7th inst., to pass a resolution condemning what the promoters described as the suggested addition of asylum attendants to the register of the Royal British Nurses' Association. Mr. George Brown (who arrived after the proceedings had been commenced) presided, and was supported on the platform by Miss Wingfield and Dr. Bedford Fenwick, the latter of whom occupied the chair until Mr. Brown arrived.

Miss Wingfield, after reading a number of lengthy letters she had received from sympathisers with the object of the meeting, read a long paper, in which she broke a lance with Dr. Outterson Wood, and others, who for weeks past have been writing to the various medical papers on this question. If, she said, the R.B.N.A. was in future going to admit mental nurses with training in special branches only, the register could not any longer be called the register of "trained nurses." In the end she proposed the following resolution :—

"That this meeting condemns the suggestion accepted by the General Council of the Royal British Nurses' Association to admit to membership, and to place upon the Register of Trained Nurses, asylum attendants who have not been trained in a general hospital, and who do not conform to the regulations for membership and registration. And this meeting considers that such a course would be both injurious to the nursing profession and dangerous and misleading to the public."

Up to this things had been deadly dull, but Mrs. Charles Hughes, who was vaguely described as "of Manchester," imparted a little life to the meeting by a brisk attack on both the proposal to admit mental nurses and those who had suggested and defended the proposal. As no reasons had been given, she said, by those who proposed the addition of asylum attendants to the register, people must look for them themselves. It seemed probable—and she emphasised the word probable—that it had originated in the desire of some well-meaning but mistaken person to obtain additional entrance fees, for it had been whispered—more than whispered—that their Association was in need of money. "Financial embarassment, as many of us know, drives people to strange devices," said Mrs. Hughes, and her audience by their applause showed a keen appreciation of the sentiment.

Discussion was then invited, but no one appeared inclined to lead the way, and it almost seemed as if the meeting was going to end abruptly, when Miss Graham stepped timidly into the breach, and urged her hearers "to vote true to their colours, and to bear in mind that a house divided against itself cannot stand," failing to see how the last part of her remark applied to the action of the promoters of the present meeting with regard to the parent association. Another lady, whom the chairman would insist on calling Miss Homerton in spite of Dr. Bedford Fenwick's audible prompting, having spoken, Miss Margaret Breay, who was rapturously greeted with applause, evidently as some sort of recompense for her recent martyrdom in the cause of free speech, deplored the fact that they had been obliged to hold that meeting that day. They ought, she thought, to have been able to bring their views before the Executive Committee and to be sure of a fair hearing. They ought not to have to appeal to the public for support against their officials. However, they were not sure of a fair hearing—in fact, they would be very much surprised if they got it. She proceeded in the same spirit to give a lengthy history of the cruel way in which the irreconcilables of the R.B.N.A. had been treated. Even Dr. Bedford Fenwick, "whose unique work for nurses will only be realised and appreciated by future generations," was, she said, prevented from speaking. That gentleman followed with a dissertation from the same standpoint which lasted for about a quarter o an hour. Then an elderly gentleman who gravely announced that he had been an inmate of various lunatic asylums for years, and was now "enlarged during Her Majesty's pleasure," commenced to give his views on mental nurses, a matter which he seemed to think his experiences qualified him in the highest degree to pronounce an opinion on. Eventually, through the soothing efforts of the Chairman and Dr. Bedford Fenwick, he was prevailed on to resume his seat, and the resolution was put and carried unanimously. Votes of thanks to the Chairman and Miss Wingfield—in the latter case proposed by Mrs. Bedford Fenwick, "for the public spirited way in which she has called this meeting together"—concluded the proceedings.

---

### Quarterly Council Meeting of the Royal British Nurses' Association.

A QUARTERLY meeting of the General Council of the Royal British Nurses' Association was held at the offices on Friday, (8th inst.), H.R.H. the Princess CHRISTIAN (president of the Association) in the chair.

The HON. MEDICAL SECRETARY (Mr. E. A. Fardon) stated that letters had been received from various members on the subject of the admission of mental nurses to the Association, amongst which was the resolution (given above) which was passed by the meeting at St. Martin's Town Hall on the previous day. He added that no formal scheme had yet been prepared on the subject.

It was decided that the letters should not be read at that meeting.

The HON. TREASURER read his report for the past quarter, and moved its adoption. This was seconded by Mr. GANT, and carried unanimously.

The HON. MEDICAL SECRETARY read the report of the Executive Committee for the quarter, and called special attention to two of the paragraphs in that report. One of these referred to the resignation of Miss Ravenhill the secretary of the Association, and he took the opportunity of expressing, on behalf of the committee, regret, not only at that resignation, but doubly so at the cause which had led to it. After speaking in eulogistic terms of the work Miss Ravenhill had done for the Association, Mr. Fardon passed on to the other paragraph, which was one relating to the question of mental nurses, He wished to refer to it because he saw by the reports in that morning's papers that a meeting on the question had been held on the previous day at St. Martin's Town Hall. It was reported that it was convened by Miss Wingfield, a member of the Council of the R.B.N.A., and amongst those present was another member of the Council, Dr. Bedford Fenwick, who made a speech which Mr. Fardon referred to at some length. He did so, he said, because he had no recollection that when this matter was before the Council, Dr. Bedford Fenwick took the opportunity of expressing his views on the subject, or that he even voted against the adoption of the report on the question. In Dr. Bedford Fenwick's speech as reported, he complained that discussion at the Association's meeting had been stifled and prevented. He (Mr. Fardon) fearlessly appealed to every member of the Council to say whether, when the report of the committee appointed to inquire into Dr. Outterson Wood's proposal was presented, there was any stifling of discussion at all. It would be in the memory of all that H.R.H. the President specially invited criticism. (The President: She did.) He would also like the Council to note that Dr. Bedford Fenwick was also reported to have referred to some idea that the Association was to be engaged in further law suits, whether on this matter of mental nurses or upon others he did not know. It was undoubtedly one of the privileges of the Association conferred upon it by the Royal Charter, that it could sue and be sued in its corporate capacity, but many of the members felt very strongly that the funds contributed by the nurses were not wisely spent in being frittered away on "frivolous and vexatious" litigation. (Applause.) The position of the Association with regard to the mental nurses' question was this—when the matter (which had been in the minds of several very prominent and eminent members for some time) was brought forward by Dr. Outterson Wood at the council meeting in July, it was felt that, as it was a very large subject on which most of the members of the council were necessarily but imperfectly informed, it would be best, and it was so decided unanimously, to refer it to a committee to consider and report upon. In October that report was received, discussion was invited, and it was accepted unanimously, authority being given to the president to obtain further information as to the training, &c., of asylum nurses. The committee had had more than one meeting, and the Executive Committee considered the information got together sufficient to warrant them in appointing a sub-committee of their body to consider the whole question. The question was a large one, involving the admission of a very large number of nurses, and he could not help thinking that the opposition had arisen from a want of understanding of what were the aims and purposes of the R.B.N.A. and of the persons who had brought that question forward. They were now waiting for the report of the sub-committee, which would be submitted to the Executive Committee, and, if adopted, would then be brought before the council.

H.R.H the President proposed the adoption of the report.

This having been seconded,

Dr. Bedford Fenwick desired an opportunity of defending himself against the attack made upon him by Mr. Fardon. Mr. Fardon said that he (the speaker) had complained that free speech had been prevented. Everybody knew that free speech had been prevented. (Cries of "No, no.") At the very last meeting a public attack was made upon him by Sir James Crichton Browne, and when he rose to reply to it he was prevented from saying a single word. Further than that members who had given notice of resolutions had been ruled out of order. (Loud cries of "Order, order," and a voice: "That has been settled in a court of law.") Certainly the question had been decided, and a jury had found a very distinct verdict in the matter. (Voice: "How about the Court of Appeal?") The Court of Appeal have given a different verdict—(laughter)—but the jury the palladium of British liberty—(loud laughter)—as it was very ironically termed.—(Cries of "Sit down," and interruption, in which the remainder of the sentence was lost.) Continuing, Dr. Bedford Fenwick said that, as regarded his not speaking against the report when it was before the Council, he had so often been requested not to speak, that he never did speak—(loud laughter, and "Oh, oh")—unless it was on a question which he understood. (Renewed laughter.) He did not understand the matter of mental nurses when it was suddenly sprung upon the Association, so he did not vote either for or against it, and he believed many other people did the same thing. The suggestion proposed to upset the whole principle on which the Association was founded, and proposed to put mental nurses on the register.

H.R.H. the President: It has not been said that they are going on our register. It is the general proposal which has been accepted and nothing more.

Dr. Bedford Fenwick: May I read the report which was passed at the last meeting?

The President: No. I know it quite well.

Dr. Bedford Fenwick: I am sure you do, madam; but that report makes a definite recommendation, which has been accepted, and which proposes that mental nurses should be admitted to the register and to the privileges of the Association under certain conditions.

The President: Exactly—under certain conditions.

Dr. Bedford Fenwick: May I read them?

The President: I don't think it is necessary. Everybody knows them.

Dr. Bedford Fenwick said the subject was a very important one both for the public and for the Association, as it went to the root of the question whether nurses put on the register of trained nurses were to be trained at a general hospital or not. The register was started for nurses trained in a general hospital, and now it was proposed that persons who had never been in a general hospital at all should be admitted.

Miss De Pledge rose to a point of order. That question was not before the meeting.

Mr. Gant said that Dr. Bedford Fenwick rose to defend himself, but he was now entering into a general discussion of the matter.

Dr. Bedford Fenwick said that he had defended himself as much as he cared to do, and he was now speaking to the report. (Interruption.) The scheme was one which would not be permitted to be carried out, it was doing a great deal of harm to the Association—(cries of "Question")—and he protested against the attempt.

Mrs. Hughes, of Manchester, and Miss Wingfield also protested against the admission of mental nurses to the register.

Mrs. Dacre Craven said that if anyone present knew what it was to have to find a trained mental attendant for a person who had suddenly become insane, and had had to go to one nursing establishment after another to get one, they would see how necessary it was that some association should compile a directory of this class of nurse. Such nurses should not infringe upon the privileges of, and should not call themselves, hospital trained nurses. When she sought an attendant for an insane person she did not want a hospital-trained nurse—the best trained hospital nurse in the world, without special mental training, was not fit to take charge of the insane. If this scheme was carried out, and she hoped it would be, mental nurses would be placed separately so that anyone wanting a nurse to attend to a sick person could look to the mental branch and choose one.

Sir James Crichton Browne said that he thought Dr. Bedford Fenwick had ably maintained his rôle of objector-general, and by the extravagance of some of his statements had imported a little humour into the proceedings. (Laughter.) As regarded the stifling of discussion at the last meeting anything that was done was done by the Council itself, for the Council as a body unanimously declined to hear Dr. Bedford Fenwick. (Applause.) Besides this no voice had been heard so frequently or at greater length in the room than that of Dr. Bedford Fenwick. (Laughter.) Without entering upon a discussion of the question, he offered, while the matter of mental nurses was under consideration, to obtain the admission into her county asylum of any nurse or matron who really wished to satisfy herself as to the work done and the kind of training received by mental nurses, promising that the greatest facilities should be given her to obtain information. He thought that many of the objections which existed at present would then be removed.

The report was formally put and carried, only Dr. Bedford Fenwick dissenting.

A vote of thanks to H.R.H. the President concluded the proceedings.

### A Persistent Libel.

THE editor of the *Nursing Record* has chosen to state that Mr. Burdett, in his paper, has said, "There are still members of the Executive Committee who remember the description given of them by Mr. Burdett during the period in which he vainly attempted to smash up the association through the medium of his paper, and who remember his graphic description of themselves as the scum of the nursing profession," &c. We were quite sure that this was untrue, but, for the satisfaction of our readers, we have searched the columns of THE HOSPITAL to discover what possible excuse there could be for saddling us with words which we never uttered. As a result we have discovered them in a letter written by a correspondent (who at that time was a sister in a large metropolitan hospital), and published in THE HOSPITAL on May 18th, 1889. We find it impossible to believe that these several sentences were quoted from memory by the editor of the *Nursing Record;* and yet, if it is the fact that while penning her statement that Mr. Burdett had said these things, she had before her the letter which we reprint below, which is headed "A Hospital Certificated Nurse writes," we can only leave it to our readers to form an estimate of such proceedings. We have always considered that one of the advantages offered by THE HOSPITAL to nurses in various parts of the world is that, by means of its correspondence columns, it provides an opportunity for the expression of the feelings of every class of nurse on every class of nursing question. Everyone who reads them knows what a variety of opinions there find expression. To pretend that an editor has said a thing which, as a matter of fact, was only said in a letter from a correspondent, shows an extraordinary lack either of intelligence or veracity. The following is the letter referred to, the sentences ascribed to us being printed in italics :—

THE BRITISH NURSES' ASSOCIATION.

A HOSPITAL CERTIFICATED NURSE writes: We are hearing a great deal now about this much-vaunted British Nurses' Association. Of course, most of what we have heard has been from its supporters, and therefore decidedly in its favour, for most of us nurses do not trouble ourselves about it one way or another, but let things take their own course. Now, however, I for one think it high time no longer idly to take it for granted that the British Nurses' Association is a perfect godsend to all nurses, but to consider carefully what this association professes to be able to accomplish, how it sets to work to carry out its proposals, whether it really will be of use to us nurses, and finally whether it will be well for one and all of us to join it without further delay.

For this purpose I will take seriatim the various statements made in a pamphlet issued by this association. This pamphlet states that the British Nurses' Association has been founded "to unite all British nurses for their mutual help and protection, and for the advancement in every way of their professional work. . . . Every person who claims to be a nurse, who engages in the work of nursing, without having been thoroughly prepared for the duties which that work involves, is likely to diminish not only the demand for them, but also the payment which they will be entitled to receive," and, moreover, it "aims, in the first instance, at bringing about a state of things in which no one will be allowed to call herself a nurse unless she has undergone a proper course of hospital training, of a sufficient length to give her an insight into the management of all kinds of illness, whether surgical or medical." So far so good, and if this Association were careful to receive only those nurses who can bring proper guarantees of thorough training, it might carry out its professions. And how does the British Nurses' Association set to work to attain this very desirable object, and endeavour to protect the unfortunate public, whom one would imagine all nurses had hitherto combined to injure? And why, moreover, should the public be excluded from belonging to the association? Is the association afraid that the public will rush in in such large numbers as to be inconvenient?

To return, the association will endeavour to secure properly trained and efficient nurses to the public by allowing that "every woman actually engaged in nursing is eligible," though just before it has been stated that "at present, when we hear that a woman is 'a nurse,' we hardly know what meaning to attach to the word; but when the association has succeeded in the attainment of its primary object, this meaning will be no longer doubtful." Here we have an association proposing to protect the public against unqualified nurses, and stating "that the British Nurses' Association guarantees that the person to whom this word 'nurse' is applied has passed through a prescribed course of study and practice, and has given satisfactory proof of having turned her opportunities of learning to good account," and, on the other hand, welcoming, as a member, anyone who has been engaged in nursing for three years, thus implying that any "Sairey Gamp" may have what the promoters look upon evidently as a much-to-be-coveted honour. Would anyone who has had anything to do practically with nurses make such an absurd blunder? And suppose that hospital nurses who have toiled through their two or three years' training, and finally obtained their precious certificate, and by their conscientious work have won the respect and goodwill of those in authority over them—will such wished to be mixed up in a sort of "hotch-potch" with *the scum of the nursing profession?* Anyone who knows anything about nursing will tell the promoters of this association that it is only quite recently that workhouses and asylums have discovered the necessity for trained nurses, and that, however zealous the authorities of these excellent institutions are, they can only work reforms slowly, and that there are many of the old order of nurses left. Nevertheless, the Benevolent British Nurses' Association demands only that all would-be members should have been engaged, in whatever capacity, in nursing for three years.

The worst of it is, that this sort of association will probably attract a large number of nurses from country hospitals, who have not the advantage of being connected with a hospital in London, and hearing nursing matters discussed, and both sides stated; so that they can have all the *pros* and *cons* of the cases before them. *Perhaps, too late, nurses will realise that the public and the authorities of the large training schools will indeed "know what meaning to attach to the words member of the British Nurses' Association"—namely, a nurse who has taken refuge in it to obtain pseudo-respectability, because she could not get it elsewhere.* What, then, does the British Nurses' Association offer its members in return for their half-crowns? A so-called certificate with a facsimile of Her Royal Highness Princess Christian's signature? And what use is this certificate to its owner? We will hope the founders of the British Nurses' Association can answer this question—no one else can! To hospital nurses it appears a mere farce—about as useful as the "V.R." over a letter-box—nay, less so, because Her Majesty's initials do guarantee that the letter-box is a properly-authorised deposit for letters, and that a properly-appointed officer will be responsible for all letters thrown into it. But does Her Royal Highness Princess Christian also guarantee that every nurse possessing the paper with a facsimile of her signature is also a properly-qualified member of the great nursing profession, and are those who presume to give the certificate properly qualified to judge whether she deserves it or not? We absolutely deny that a self-constituted association like the British Nurses' Association is fit to grant, from second-hand information, a certificate to any nurse who applies for it, and can pay her half-crown. Truly, if this is so, and this passport can be so easily obtained, there is no more call for the careful and systematic training given to their nurses by the large hospitals. The British Nurses' Association will kindly relieve them of all trouble and responsibility. Nurses, after three years' nursing in whatever capacity, have only to pay down their money, and they can defiantly wave the magic certificates before the face of a helpless public. Everyone knows how many women apply at hospitals to be trained as probationers, what a number of these fail, or have to be dismissed, as unsuitable before the end of their training, but because they have been in a hospital for a short time they think they can be private nurses. Such are the women who, we venture to prophesy, will rush into such an association. And we ask any impartial judge, are such women calculated to raise the status of the nursing profession? If such people make up the members of which the British Nurses' Association announces itself to be composed, we wish it joy of its members. And far from regretting, as the pamphlet does, that the authorities of a few public institutions have advised their nurses not to join the British Nurses' Association; we, with many other hospital-trained nurses, regret *very much to hear that any institutions have shown so little regard for their nurses' true welfare as not to have used every effort in their power to prevent their joining so hurtful an association.* The British Nurses' Association has evidently been formed to drag the nursing profession into a prominence not desired by the best nurses, who treasure most the respect and goodwill of their *alma mater*, where their powers are ruly appreciated.

### The Johannesburg Hospital.

In consequence of information which we have received from Johannesburg, we feel bound to warn nurses most emphatically against all thought of undertaking hospital work there until the present difficulty is thoroughly cleared up. As we stated last week, several of the nurses who recently went out to the Johannesburg Hospital have been fined £100 each for breach of contract. Having, as they state, found the conditions of the service quite different from what they had been led to expect, and, to put the matter to the test, three of them left the hospital, with the result above mentioned. The following comments on the case we quote from the *South African Review*:—

"Some time ago the Johannesburg Hospital Board engaged a number of nurses in England to proceed to the Rand. They had to sign a contract by the provisions of which they agreed to forfeit the sum of £100 (each) should they not adhere to it, or leave the service of the Board, for any but adequate reasons, before the expiry of the period agreed upon. These nurses on arriving at Johannesburg soon found that they had to 'pig' together—not to put too fine a point on it—and that any regulations or conditions to ensure a reasonable amount of comfort were conspicuous by their absence. Three of the nurses, by way of bringing a test case before the courts, left the hospital and were sued for £100 damages for breach of contract. It transpired that they were only receiving from the Board £4 12s. per month, a salary which is outrageously small, that they were compelled to be on duty for nearly twelve hours on end, only at the close of their toil to repair to a bedroom in which they were all compelled to herd together. It also transpired that they were only allowed two meals a day. The case was heard in the court of the second Judicial Commissioner. The first Commissioner, Mr. De Beer, being also a member of the Hospital Board, the case was, naturally, not heard by him. But it soon became apparent, during the hearing of the case, that Mr. De Beer might just as well have been on the Bench, for he sat in the body of the court, interpolating remarks as the case proceeded, and in fact displayed such bias against the nurses that his subordinate, the Second Commissioner, practically had no option but to give judgment for the Board. These poor nurses are, therefore, mulcted in damages to the tune of £100 each, and their colleagues at the hospital must perforce put up with the bad treatment of the Board or share a similar fate. These facts cannot be too widely known by nurses in England. Any of their number who may contemplate proceeding to the Rand under a contract with this Board will do well to reconsider their plans. Once in the hospital there they are practically slaves, and redress in the courts is difficult to obtain because the officials and legal luminaries at Johannesburg are hand in glove with the Board, and are concerned more in ensuring that the Board shall obtain its money's worth out of the nurses rather than that the nurses shall be fairly and properly treated under their contracts. I trust that this case may come under the cognisance of the nursing institutions at home, and that some steps may be taken on behalf of the unfortunate members of the profession now undergoing shabby treatment at the Johannesburg Hospital."

These details are corroborated, and others are added by the *Critic*, a paper published at Johannesburg. It will be remembered that Miss Young and a staff of thirty nurses, who had been selected from leading hospitals, left England on January 4th last year, on the faith of statements that the hospital to which they were proceeding was a well-equipped institution, which would compare favourably with many hospitals in England, and of an agreement that they would have ample accommodation, separate rooms, good food, and attendance, and £55 a year salary. It is to be borne in mind that the value of this salary is to be estimated in accordance with the value of money in Johannesburg, and that the papers there speak of it as "a Kaffir wage," and it also should be mentioned that two of the nurses who left England a year ago have already died, and that it was stated at the trial that they died from overwork and want of attention. In any case, however, the nurses maintain that they were unfairly treated, that, except the wages, the conditions on the strength of which they were led to go out have not been observed; and that, lastly, they have been unfairly tried. A correspondent has raised the question whether the contract which was signed in England ought to have been signed afresh on arriving in the Republic. Be that as it may, we would warn nurses who may be asked to go to Johannesburg to take good legal advice as to the responsibilities they are incurring before they sign any contract, and to bear in mind that in such a climate it is quite impossible in the summer months to work long hours. Any undertaking to do the same work as in England may readily be turned into a bond of slavery.

### Appointments.

### MATRONS.

Garston Infectious Diseases Hospital.—Mrs. Eliza Mann has been appointed Matron at this hospital. Her previous work has been as auxiliary nurse at Stockport Isolation Hospital, matron for the Shelter of the National Society for the Prevention of Cruelty to Children, nurse in surgical wards of the Manchester Royal Infirmary.

North-Western Fever Hospital.—We regret that in announcing Miss Maud M. Lloyd's appointment as Matron of this hospital last week the name of the Royal Infirmary, Liverpool, was given in mistake for the Mill Road Infirmary, where Miss Lloyd has been assistant matron for some time past.

Sheffield General Infirmary.—Miss Dorothy K. Ruston has been appointed to the Matronship of this hospital. She was trained at the Royal Southern Hospital, Liverpool, where she subsequently held the posts of night sister, sister of the children's ward, and sister of the male surgical wards, the last for over five years. For the last two years Miss Ruston has been matron of the Home for Incurables, Liverpool. Her testimonials are excellent.

### Where to Go.

Royal Institute of Painters, Prince's Hall, Piccadilly.—A café chantant in aid of the Victoria Home for Invalid Children, Margate, under the immediate patronage of H.R.H. the Duchess of York, will take place at the Galleries of the Royal Institute of Painters, Piccadilly, on Saturday, January 23rd, at three p.m. The committee have arranged a very attractive programme. The tickets (including refreshments, supplied by Benoist) are three shillings each, and may be obtained from the hon. secretary, 8, Ashley Place, S.W.

### Death in Our Ranks.

Miss Alice F. C. Hamilton, granddaughter of Mr. James Hamilton, of Fintra, co. Donegal, died suddenly at the City of Dublin Nursing Institution, of acute peritonitis, contracted while nursing a private case on January 3rd.

### Hospital Entertainments.

### St. Mary's Hospital.

On Wednesday and Thursday in last week the resident medical officers at St. Mary's Hospital gave a very good dramatic entertainment to the patients, nurses, and their friends. The programme consisted of two farces, the ever-popular "Area Belle" and "My Turn Next," with a miscellaneous concert during the interval of half an hour between the pieces. In the latter play Mr. Marshall was most amusing as Teraxicum Twitters, the village apothecary, while Mr. E. G. Sworder and Mr. A. W. Sanders were excellently made up as Lydia (Twitter's wife) and Cicely (her sister), carrying out the parts very well. The sight of "our doctors" thus disguised was a source of great amusement and delight to the patients, and the performance was much enjoyed by everybody.

### Paddington Green Children's Hospital.

The annual treat and entertainment to the little patients at the Paddington Green Children's Hospital was given on Wednesday, the 6th inst. A Punch and Judy show and a magic lantern display delighted the children, and a great excitement was the pulling of a huge cracker, twelve feet long, filled with toys and presents, to be afterwards distributed. The wards were prettily decorated with ribbons by Miss Anderson and her nurses.

### Royal Free Hospital.

An entertainment, organised by the resident medical officers of the Royal Free Hospital, Gray's Inn Road, was held in Calthorpe Ward on Thursday afternoon last. The ward, which is the one set apart for the treatment of the many accidents received at this hospital, was very prettily decorated with flags, banners, and Chinese lanterns, and between the beds and in every available corner seats were placed for the accommodation of the convalescent patients from all the other wards. This number was augmented by the nurses, members of the staff, students, and a few other friends, who together formed—as the performers found as the repeated demands for encores were made—a highly appreciative audience. Amongst the ladies and gentlemen who kindly gave their services were the Misses Clifford and Rowe, and Messrs. R. Selbie, Clayton, and Ring, of the Gaiety Theatre. A "baby talk" by the last-named gentleman was delivered with such telling naturalness as to evoke acquiescing responses from several of a group of tiny patients, who, with heavily bandaged heads and limbs, occupied privileged seats immediately beneath the stage.

### London Association of Nurses.

A New Year's "At Home" for members of the London Association of Nurses was held at 3, Burwood Place on January 7th. There was a large gathering of nurses, who greatly enjoyed meeting the superintendent, and many old friends, and a very pleasant afternoon was spent. Tea and conversation were interspersed with music, songs, and recitations.

### City Hospital, Park Hill, Dingle, Liverpool.

On Thursday evening last the nurses of the City Hospital, Park Hill, gave an entertainment, consisting of a short play entitled "Britannia's Tea Party," and tableaux. The nurses who took part in the play adopted the national colour for their dress, thus representing the different countries. The tableaux were very successful, and the nurses entered into the evening's enjoyment with right good will, and appreciated the kindly interest shown by their matron, Miss Givins, and the resident medical officer, Dr. Kennan, in their evening's pleasure. Dr. Robinson, late resident medical officer of Park Hill, helped to make the evening more pleasant by his musical abilities, and songs were sung by the nurses. The limelight was lent and worked by Mr. Lee, and the nurses thanked him specially for his great kindness.

### The North Lonsdale Hospital.

The patients at the North Lonsdale Hospital spent a very enjoyable Christmas. The wards were charmingly decorated. Bright wreaths of holly, mistletoe, and other evergreens festooned the walls, green muslin curtains draped the windows, plants and flowers were placed up and down the ward, and in the centre of the floor a gigantic Christmas tree reared its head to the ceiling. Santa Claus brought the children well-filled stockings, while the grown-up folks found cards left by the mysterious midnight postman, who visits hospital wards on Christmas Eve. At a quarter to ten a.m. a bright Christmas service took place in each ward, and at half-past twelve p.m. dinner was served in the wards, the matron aad house surgeon dispensing the turkey, goose, plum pudding, and dessert, so liberally provided by the many kind friends of the institution. After dinner the smokers were allowed to light up the pipes and cigars with which they had been so plentifully provided. At four p.m. a number of ladies and gentlemen arrived, and the rest of the evening was devoted to all sorts of entertainments, the patients each being allowed to invite a friend to tea at half-past six.

### Everybody's Opinion.

[Correspondence on all subjects is invited, but we cannot in any way be responsible for the opinions expressed by our correspondents. No communication can be entertained if the name and address of the correspondent is not given, or unless one side of the paper only is written on.]

### BED MAKING.

Superintendent writes: May a district nurse, who has always used an under blanket when she could get one, suggest that they only tend to produce bed sores when improperly used; that is, when put, as they often are, next the patient. When a patient is quite helpless, and requiring a large macintosh, it does not much matter whether he has a blanket under it or not; but if no macintosh is needed, most nurses will agree that both patient and mattress (or bed) are the better for having one.

### SUPPORTED BY VOLUNTARY CONTRIBUTIONS.

"An Old Nurse" writes: It has puzzled me lately what "supported by voluntary contributions" really means, and why several institutions for nursing the sick poor, which receive some of their income from the rates and taxes, still try to delude the charitable public into the belief that they are voluntarily supported hospitals and nursing institutions in order to get their voluntary offerings. Surely a voluntary donation must be one in which the donor can give any sum he wishes and only when he wishes. Now, money taken from the poor rates and given by the guardians to any charitable institution cannot be a voluntary contribution. If the guardians of a district, acknowledging the good work done by such institutions, volunteered to give a year's contribution out of their own incomes such a gift would truly be a voluntary contribution and bless both the givers and receivers. But money taken from the poor-rate payers (many of whom are quite poor) by force surely no reasonable creature can count a voluntary gift. We do not count union infirmaries voluntary hospitals, neither should we count institutions which receive some of their income from the rates voluntary supported ones. Would it not be better to let all the rates which can be procured go towards improving the well-being of the poor sufferers in the union infirmaries and keep the good old purely voluntary system for those noble and philanthropic institutions outside the unions of which England for so many centuries has been so justly proud? This small contribution which several institutions take from the rates is, I fear, the "thin end of the wedge"; and if this system goes on and increases all our voluntary hospitals and nursing associations will be robbed of their highest influence for good on the poor, and finally merge into rate-supported institutions or union infirmaries. And that, I am sure, not only the charitable public, but those deeply interested in our noble voluntary institutions would greatly deplore.

*** Our correspondent seems to be making herself unhappy over a very imaginary evil. The contributions of the guardians must be looked upon as payment for work done or to be done. On no other ground would they be passed by the auditor. So far as nursing is a necessity, the guardians are bound to provide for it. In many cases the patients can be quite as well and more comfortably nursed at home, while at the same time they are entitled to help from the rates, which they can only get by going into the infirmary, the last thing the self-respecting poor will do until compelled. The district nurses, by caring for such cases, are relieving the rates of a burden, and where this happens to any extent it is only right and fair that the guardians should devote a portion of the money entrusted to them for the good of the poor in helping them in this indirect but most efficacious way. So far from objecting to this proceeding, it is one to be much encouraged, and we cannot see anything to alarm the most devoted supporter of our excellent voluntary system in these contributions to district nursing associations. They give the guardians no control over the management of the institutions beyond that of ordinary subscribers.—Ed. *T. H.*

### THE R.B.N.A. AND MENTAL NURSES.

Dr. A. R. Urquhart, Medical Superintendent of the Murray Asylum, Perth, writes: I have read and re-read your article on mental nurses in last week's issue of The Hospital, and still I wonder. There is, indeed, cause for astonishment. The Hospital professes to be the organ of the nursing world—to do the best for hospital administration irrespective of the special work done in our medical institutions. In face of the efforts being made to equalise these institutions, it seems to me that the tendency of your article is to maintain the antiquated and obstructive barriers which have taken so much time and energy to pull down. All nurses should be registered. I emphatically object to the trained-hospital nurse objecting to the trained-asylum nurse being placed on the same level. If invidious comparisons as to importance are to be instituted, the balance dips the other way. We are fast emerging from the bad old times, and the service is attracting the same class as besiege the doors of the general hospitals. I note that you limit your experience to "some knowledge of an asylum." It is certainly not the Royal Edinburgh Asylum, where there are four "hospitals" in connection with the institution through which all attendants and nurses pass in the course of their training. Such a system is indeed common property, although there are in this, as in every similar instance, notable exceptions. I desire specifically to state that no amount of cramming from a text-book will suffice for the examination of the Medico-Psychological Association. Practical instruction in the wards is the main feature of the necessary training. That is supplemented by lectures, demonstrations, and the handbook in use. There must first of all be proved suitability; manual dexterity and mental instruction follow. I have acted as examiner and as assessor over and over again at these examinations, and can unhesitatingly declare that in my experience no attendant or nurse will obtain the certificate without a thoroughly competent knowledge of the care of the sick in body and mind. It is not the intention of the association to breed a partially-informed race of doctors. It is their aim to develop nurses in the best sense of the term. I rub my eyes when you lend the weight of your influence to the perpetuation of half-measures—hospitals training their own nurses and giving their own certificates. Has the medical profession, indeed, travelled so far as to secure a minimum training and a minimum degree of knowledge amongst those who would join the ranks, and are we to stop short in regard to the nursing profession? Just as there were medical schools of low standard and of high standard, so there are hospitals of easy morals and of rigid virtue. Free competition will not insure all the gifts and all the graces; no, nor a decent standard of knowledge—else would American notions in University degrees be truly desirable. Contrariwise, we require that the evils of free competition should be minimised by judicious restraint. I am not prepared to say whether nurses should first enter a general hospital or an asylum if their ambition is to gain the higher appointments open to their profession. In my experience we have had nurses from hospitals entering on asylum work, and nurses going to hospitals after service here, in the pursuit of their vocation. While this question was still in its infancy, I determined to prefer as matron a nurse of large hospital experience, and had a lively correspondence with men of eminence in the asylum world relative to this point. One distinguished physician wrote to me his opinion that, "the mental attitude and practical training of a nurse in a general hospital would not usually conduce to her success in an asylum." There is some truth in this proposition. It is so firmly imbued in the minds of some that hysteria is a manifestation to be firmly repressed, and that mental disease is really in essence hysteria, that they have been thereby incapacitated from doing the best possible. Other objections might be started; but I shall not tresspass on your space in regard to this view, which is not borne out sufficiently to become a guiding rule in practice. The truth is that the successful matron is born, not made. She will use opportunities, and gain experience, when others will fail to mark, learn, and inwardly digest. The committee did not seek to limit all candidates for the higher posts in asylums to those who hold the certificate they instituted; but there are occasions on which the certificate would turn the scale in favour of its owner; and the time may come when the nursing profession is sufficiently organised to apply such a rule, just as the British public is in some degree protected by the registration of medical men under the medical Acts That is a large question, but not so far off, perhaps, as might appear. In the meantime the Medico-Psychological Association are well advised in doing all that is possible to carry the standard high. Your last paragraphs, appropriately enough, carry the sting of your remarks. Can an enemy have done this? You advise the Medico-Psychological Association to limit its endeavours to social functions and questions of pensions—the loaves and fishes—nay, the remainder biscuit of the voyage on which they set out, more than fifty years ago. One must not say anything unseemly of an editor in his own newspaper; but, sir, do you think it likely that your counsel of perfection will be followed? The objects of that association are, first of all, the promotion of science in relation to mental disorder; then the improvement of the treatment of the insane; and, last of all, good fellowship. So far as I know the members, they are not the men to hand over their high office to the keeping of any other society; and I can speak with equal respect of the British Medical Association, as for years a member of the council of each. I have never observed that the portals of the one are more sacrosanct than those of the other; and only learned at Carlisle what an inept, villainous, and contemptible sort of creature went to make up the council of the British Medical Association. I had not observed it before; but the Breays are always with us, and they tell us. My only recollection of British medical opinion in this matter is the adverse criticism of nursing certificates from certain hospitals—not asylums. How runs the blessed catch-word? The establishment of an inferior order of medical practitioners, or words to that effect. I yield to no asylum physician in my sense of the value of the meetings of the British Medical Association, whether general or local. They bring specialists into contact with the main body of their profession, and widen the outlook for us all. I have yet to learn that the British Medical Association is in a position to aid us in the matter now under review. At any rate, Heaven helps those who help themselves, and it will not do for us to call upon Jove without putting shoulders to the wheel. The car moves slowly; but it is going in the right direction. Mind you do not get in the way of Juggernaut.

*** It is not a question of registration. We yield to none in our wish to raise the status of the asylum nurse, but for her own sake, and in justice to the hospital nurse, with whom she is to stand on an equality, we must insist upon a period of training in the sick-room as a necessary part of her education. It is because we do not wish nurses to remain with a minimum degree of knowledge that we take this standpoint. A mental attendant is one thing, and may be efficient without hospital training. But if we are to use the title of mental nurse, it must be strictly confined to those who have had an adequate hospital training as nurses in addition to the asylum experience. Limitation of space forbids publication of other letters, which we have received from Dr. P. MacDonald and Mr. Ernest White, M.R.C.P.—Ed. *T. H.*

### A Book and its Story.

### THE STORY OF MAURICE LESTRANGE.

"MAURICE LESTRANGE," Mr. Omond tells us, in the preface to his novel,* "is supposed to have written his narrative more than thirty years after he left Scotland; but it is believed that the account which he gives of the condition of that country and of the state of society there at the time of his visit is accurate in most respects."

The writer, we venture to say, is on fairly safe ground when he thus challenges criticism, on a subject of which he shows himself to be so complete a master. There is no doubt whatsoever to the most casual readers of this book that the author is an authority on the particular period of history with which he deals in these pages, and on which he brings the "specialist's" mind to bear.

To the general public the merits of a historical novel depends upon the effect produced thereby upon the reader's mind. Has the picture presented by the writer the ring of truth and reality about it; do we move in the scenes he depicts, and feel that the spirit of the age, which he describes, has gained a mastery over our senses? If so, the novel is a good one and has done its work. for a real effect was never yet produced by unreal methods, Of a portrait, painted by a master hand, we can say it *is* a portrait, and qualify it according to our enthusiasms, even when the original with which to compare the picture is not actually at hand. And our criticism is justifiable. As in art, so in literature—the same method holds good. Historical ver similitude is to be gauged in a like manner; and laying on one side matters of technical comparison, we can say of a canvas as we can of a book, it is a good portrait.

And the picture of the eighteenth century, as shown by the writer in the volume under discussion, has the merits which classify it under the heading of a good work of art as above defined. "Maurice Lestrange" is a living person to us, as, indeed, are the other characters with whom he is thrown in contact, and his travels and adventures maintain their interest unflaggingly throughout his autobiography. The hero of this story is a Frenchman, the grandson of one of Viscount Dundee's officers, who had left Scotland at the Revolution and gone to France. This officer, Patrick Arbuthnot by name, married a rich French widow, took her name of Lestrange, and lived at Lachasse, her estate in the North of France, which, at the time this story opens, had become the property of Maurice Lestrange.

Maurice pays a visit to Scotland in the winter of 1764-65; and, while passing through London on his way to the North, meets his cousin Francis Arbuthnot, a captain in one of the newly-raised Highland Regiments, who is on his way home from India, having been invalided by sunstroke. The cousins travel together to Edinburgh, where, on the night of their atrival, Maurice Lestrange meets Alison Carnegie, the heroine. While dressing for a ball he hears the sound of a violin in the next room, on entering which he witnesses the following scene: "The window was thrown wide open, and close to it Arbuthnot was standing, dressed in a rich coat of blue velvet, blue satin breeches, and black silk stockings. He had gilt buckles on his shoes, wore a full-dress wig, and had altogether a very fine appearance. On a chair near him lay his hat and sword. He was playing the music of a minuet, and as he played he looked, from time to time, through the window, to where, on the opposite of the narrow close, another window was open, through which I beheld, in a brilliantly-lighted apartment, the figure of a lady dancing—a vision of brown hair, brushed back from the forehead, as was the mode at that time, and slightly powdered, of smiling lips, and grey eyes sparkling as she moved, holding up her dress on either side, in the steps of the minuet. Thus, for the first time, I looked upon the loveliest woman I had ever seen."

That evening he meets Miss Carnegie at the ball, and a friendship is struck up between them, which, on the part of Lestrange, at least, soon ripens into love. After a stay of a few days in Edinburgh, during which he is hospitably entertained, and sees a good deal of the curious customs of that hard-drinking, hard-thinking capital, Lestrange departs, in company with Francis Arbuthnot, for Westermains, in Perthshire, the family property of the Arbuthnots, and before they have been there very long it comes out that Thomas Arbuthnot, the "Laird of Westermains," is going to marry Alison Carnegie, who has been persuaded to make the marriage by her family, who regard Thomas Arbuthnot as a suitable husband, although Alison does not even pretend to any other feeling but that of friendship towards him.

Lestrange continues his travels, from Perthshire to the North of Scotland, visiting the battlefield of Killiecrankie, at which his grandfathers had fought; and there is a short description of the condition of the Scottish Highlands, which were then almost a *terra incognita* to Englishmen, and still more to foreigners like Lestrange. On his return from the North, he once more visits the household of the Laird of Westermains. The marriage has already taken place, and soon after Lestrange, perceiving that things are not going very smoothly, obtains from the bride a promise that, in any trouble she will send for him. Unfortunately, at this time a relation of the Arbuthnots, Bella Kennedy, is on a visit to Westermains. This woman, hypocritical, ill-tempered, and unscrupulous, has a prospect of succeeding to the estate, and she sets to work to make the laird jealous, by insinuating that his wife and Francis Arbuthnot are deceiving him. This subject, as well as some difficulties about money, leads to a violent quarrel between the brothers, and Lestrange leaves Westermains with Francis. A week later the former, who is now about to return to France, receives a sudden summons from Mrs. Arbuthnot, begging him to return to her house. The laird has died suddenly, and she is in trouble. He starts at once, and the plot thickens from this point until, on the day fixed for the laird's funeral, Francis Arbuthnot and Alison are arrested on the charge of having conspired together to poison the dead man. Lestrange, who has now almost avowed his love to Alison, follows the prisoners to Edinburgh, where they are tried before the High Court of Justiciary, on the charge of murder. The whole account of this trial, as indeed the circumstances immediately surrounding it, shows that the writer has something more than a passing acquaintance with the technicalities of Scottish law. The legal mind betrays itself in every well-considered line, and much of the value of the book centres round this climax in the story. The result of the trial is a verdict of "Guilty;" but Lestrange manages, with the assistance of a strange being, a cripple, who figures in the early pages of the book, and who was indebted for certain kindnesses at the hands of Maurice and Francis, to effect the escape of Alison. The proceedings of this escape are forcibly brought before the reader's eye, but sensational as is the episode it is commendably treated by the author with a certain artistic restraint. Upon the successful issue of the plot to rescue Alison, she leaves the country with her rescuer, Maurice, and goes to France, where the two are happily married.

Francis Arbuthnot, whose part in the dismal tragedy, the main outlines of which are, we believe, to all intents and purposes, true, it would take too much space to narrate, dies of an overdose of laudanum on the night before he was to have been executed.

* "The Story of Maurice Lestrange." By G. W. T. Omond. (London: A. and C. Black. 1896.)

### For Reading to the Sick.

GOD'S SERVICE.

**Verses.**

NOT one minute in the year,
But I'll mind Thee;
As my seal and bracelet here,
I will bind Thee.
In Thy Word, as if in heaven,
I will rest me;
And Thy promise, till made even
There, shall feast me. —*H. Vaughan.*

He lives who lives to God alone,
And all are dead beside;
For other source than God is none
Whence life can be supplied.
To live to God is to requite
His love as best we may;
To make His precepts our delight,
His promises our stay.
Can life in them deserve the name,
Who only live to prove
For what poor toys they can disclaim
An endless life above. —*Cowper.*

The Christian knows his time is short,
But oh! the way is rough and drear;
And bowers of bliss are nigh to court
His spirit from its high career.
Let him not swerve, for storms and night
The erring soul have oft opprest;
But who hastes on is sure of light
To guide him to his promised rest.
—*John Keble.*

It may be in the evening,
When the work of the day is done,
And you have time to sit in the twilight
And watch the sinking sun,
While the long bright day dies slowly
Over the sea,
And the hour grows quiet and holy
With thoughts of Me.
While you hear the village children
Passing along the street,
Among those thronging footsteps
May come the sound of My feet.
Therefore I tell you, Watch!
Let your door be on the latch
In your home,
It may be at midnight
That I will come. —*B. M.*

God's service is our most important, if not our sole work. The spirit in which we serve Him should be entirely without reserve. Have we no reserve with Him now? Is there really no corner of our heart over which He is not absolute Lord? Does He ask of us freely what He wills, and do we do our best to give him all that He asks? Have we no implicit bargain or condition with Him that He is only to go so far with us and no farther? Is our outward life utterly and unconditionally dependent on Him? And further, is the kingdom of our inward intentions reposing peaceably beneath His unquestioned sceptre?—*F. W. Faber.*

Think nothing too little; seek for the Cross in the daily incidents of life; look for the Cross in everything. Nothing is too little which relates to man's salvation, nor is there anything too little in which either to please God, or serve Satan.—*Dr. Pusey.*

With bowed heads and open hearts may we offer ourselves. We can do no more, and we dare do no le[illegible].—*Westcott.*

### Minor Appointments.

STOCKPORT ISOLATION HOSPITAL.—Miss M. D. Bryan has been appointed Charge Nurse at this hospital. The notice we have received does not state where Miss Bryan trained. Her previous appointments have been at the Cork Street Fever Hospital, Dublin, at the Liverpool City Hospital, and at the Birmingham City Hospital.

### Notes and Queries.

The contents of the Editor's Letter-box have now reached such unwieldy proportions that it has become necessary to establish a hard and fast rule regarding Answers to Correspondents. In future, all questions requiring replies will continue to be answered in this column without any fee. If an answer is required by letter, a fee of half-a-crown must be enclosed with the note containing the enquiry. We are always pleased to help our numerous correspondents to the fullest extent, and we can trust them to sympathise in the overwhelming amount of writing which makes the new rules a necessity. Every communication must be accompanied by the writer's name and address, otherwise it will receive no attention.

*Hospitals and Christianity.*

(88) Are there any hospitals for the sick in the world built by other than Christian sects or religions?—*T. H. D.* Stamped envelope for reply.

Read the notice heading this column concerning replies by letter. You will find the fullest information respecting hospitals from the earliest times up to the present day in Burdett's "Hospitals and Asylums of the World" (which you should be able to see at a reference library), or in "Medical History from the Earliest Times," by E. T. Withington (Scientific Press, 28 & 29, Southampton Street, Strand, London, W.C.).

*Children's Nurses.*

(8[illegible]) Kindly tell me through your columns the address of the institution in London where ladies are trained as children's nurses?—*F. P.*

The Norland Institute, 29, Holland Park Avenue.

*Uniform.*

(90) I wish to alter the uniform of my nurses, and have seen in another hospital a dress I should like to adopt. Can I do so without permission from the institution in question?—*Matron.*

There is nothing to prevent your adopting any uniform you please for your nurses, except as a matter of etiquette. Most hospitals like to keep a distinctive dress so far as the limitations of uniform will allow, and a fellow feeling on the subject usually keeps matrons from copying too exactly, unless they know that this "sincerest form of flattery" would not be objected to. Can you not make some slight difference either in colour of material or way of making, which would give you what you want without being a precise duplicate?

*District Work.*

(91) Where can I get training in district nursing? I am 33.—*L. D.*

You do not say if you have already had training in general nursing? If not, you should set about that first and enter a general hospital at once, if possible for not more than two years, the district training to follow. If you have already trained in general nursing, write to Miss Gray, the Superintendent of the Metropolitan Nursing Association, 23, Bloomsbury Square, and ask her advice, enclosing stamped envelope for reply.

*Posts Vacant.*

(32) Please say if it is usual to appoint a matron without advertising or giving public notice, as has just been done at the Rotunda Hospital, Dublin? Are not such appointments usually offered for competition?—*Would-be Candidate.*

There is no reason whatever to oblige an institution to advertise the post of matron as vacant, if the right person is to be had without going to that trouble and expense. If the committee of a hospital or any other institution are able to find a suitable matron without making the appointment public, and do so, no "would-be candidate" has any right to feel aggrieved in the matter merely because they would have liked to have the post themselves.

*Stewards.*

(93) Is it usual for the large steamboats to have a special male attendant for the sick? If so, can you give me particulars, or tell me to whom to apply for information?—*Male Nurse.*

The stewards attend upon sick passengers, and many of the big liners take trained nurses as stewardesses. If you have had experience in nursing it might be a useful qualification in obtaining a post as steward; and you should write to the offices of the steamship companies, P. and O., Cunard, &c.; you will find the addresses in the daily papers.

*Homes for the Aged.*

(94) Will you kindly tell me if there is a home for the aged anywhere in the Midlands?—*Grannie.*

We do not know of any. There are numbers of almshouses scattered over the country which might answer your purpose; but you do not say if the home is wanted for man or woman, or in what class of life, or if payment can be made, so it is impossible to reply very fully. You will find complete lists of charities in Burdett's "Hospitals and Charities," on p. 790, and you might obtain some information by writing to the Hon. Secretaries of the Homes for the Aged Poor, 5, Grandacre Terrace, Anerley, S.E., or the Aged Pilgrims' Friend Society, 83, Finsbury Pavement, E.C. These institutions have their homes in or near London.

*Massage.*

(95) I want to learn massage. Can you tell me what the training would cost?—*E.S.*

We cannot answer queries which are unaccompanied by the writer's [illegible]ame and address.

